# Advancements in Periodontal Regeneration: A Comprehensive Review of Stem Cell Therapy

**DOI:** 10.7759/cureus.54115

**Published:** 2024-02-13

**Authors:** Tanvi Bharuka, Amit Reche

**Affiliations:** 1 Dentistry, Sharad Pawar Dental College and Hospital, Datta Meghe Institute of Higher Education and Research, Wardha, IND; 2 Public Health Dentistry, Sharad Pawar Dental College and Hospital, Datta Meghe Institute of Higher Education and Research, Wardha, IND

**Keywords:** personalized medicine, clinical trials, immunomodulation, regeneration, stem cell therapy, periodontal disease

## Abstract

Periodontal disease, characterized by inflammation and infection of the supporting structures of teeth, presents a significant challenge in dentistry and public health. Current treatment modalities, while effective to some extent, have limitations in achieving comprehensive periodontal tissue regeneration. This comprehensive review explores the potential of stem cell therapy in advancing the field of periodontal regeneration. Stem cells, including mesenchymal stem cells (MSCs) and induced pluripotent stem cells (iPSCs), hold promise due to their immunomodulatory effects, differentiation potential into periodontal tissues, and paracrine actions. Preclinical studies using various animal models have revealed encouraging outcomes, though standardization and long-term assessment remain challenges. Clinical trials and case studies demonstrate the safety and efficacy of stem cell therapy in real-world applications, especially in personalized regenerative medicine. Patient selection criteria, ethical considerations, and standardized treatment protocols are vital for successful clinical implementation. Stem cell therapy is poised to revolutionize periodontal regeneration, offering more effective, patient-tailored treatments while addressing the systemic health implications of periodontal disease. This transformative approach holds the potential to significantly impact clinical practice and improve the overall well-being of individuals affected by this prevalent oral health concern. Responsible regulatory compliance and a focus on ethical considerations will be essential as stem cell therapy evolves in periodontal regeneration.

## Introduction and background

Periodontal disease is a prevalent oral health condition characterized by inflammation and infection of the structures surrounding the teeth, including the gums, periodontal ligament (PDL), and alveolar bone [1]. It is primarily caused by bacterial plaque and tartar accumulation, leading to the destruction of the supporting tissues of the teeth. The consequences of untreated periodontal disease can be severe, ranging from tooth loss to systemic health complications, highlighting the significance of addressing this condition effectively [2].

The existing treatment modalities for periodontal disease, such as scaling and root planing, antibiotic therapy, and surgical interventions like flap surgery and guided tissue regeneration, have demonstrated varying degrees of success in controlling the progression of the disease [3]. However, these approaches often exhibit limitations, including inconsistent outcomes, potential post-operative complications, and an inability to fully restore the damaged periodontal tissues to their original form and function [4].

Stem cell therapy has emerged as a promising approach for regenerating damaged periodontal tissues. By harnessing the regenerative potential of various types of stem cells, researchers aim to facilitate the reconstruction of periodontal structures, including cementum, PDL, and alveolar bone. Stem cell therapy offers the possibility of addressing the root cause of periodontal disease, presenting a novel avenue for more effective and long-lasting treatment strategies [5].

This comprehensive review aims to critically examine the advancements in periodontal regeneration with a particular focus on the potential of stem cell therapy. By elucidating the current understanding of periodontal disease, evaluating the limitations of existing treatment modalities, and exploring the emerging role of stem cell therapy in periodontal regeneration, this review seeks to provide valuable insights into the prospects and challenges of integrating stem cell-based approaches into clinical practice.

## Review

Stem cell biology and types relevant to periodontal regeneration

Overview of Different Types of Stem Cells

Embryonic stem cells (ESCs) are derived from the inner cell mass of early-stage embryos. They possess the unique characteristic of pluripotency, meaning they can differentiate into virtually any cell type in the human body [6]. This remarkable ability makes them a powerful tool for regenerative medicine, holding the potential to repair and replace damaged tissues and organs. However, their application in clinical settings could be improved by ethical and practical concerns, primarily related to the use of human embryos, raising significant ethical dilemmas and controversies. As a result, the use of ESCs in research and clinical applications is subject to strict regulations and oversight [7].

Adult stem cells, in contrast to embryonic stem cells, are found in various tissues throughout the body, where they play a crucial role in tissue maintenance and repair. Within periodontal regeneration, two prominent adult stem cells are mesenchymal stem cells (MSCs) and hematopoietic stem cells (HSCs). MSCs, found in bone marrow and other tissues, can differentiate into various cell types, including those relevant to periodontal tissues such as bone and PDL. HSCs, primarily found in bone marrow and blood, generate various blood cell types. These adult stem cells are more readily accessible and ethically uncontroversial than ESCs, making them a favorable option for research and potential clinical applications in periodontal regeneration [8].

Induced pluripotent stem cells (iPSCs) are reprogrammed from adult cells, such as skin or blood cells, to exhibit properties similar to embryonic stem cells, including pluripotency. The process of reprogramming involves the introduction of specific genetic factors that enable the adult cells to regain pluripotency, essentially mimicking the behavior of ESCs [9]. iPSCs offer the potential to generate patient-specific stem cell lines, providing a means to develop personalized regenerative therapies tailored to individual patients. This personalized approach holds promise for advancing precision medicine, where treatments can be customized to each patient's specific needs and genetic background, potentially improving the efficacy and outcomes of regenerative therapies. While ethical concerns related to iPSCs are less pronounced compared to ESCs, ensuring the safety and efficacy of iPSC-based therapies remains a critical consideration in their continued development and application in clinical practice [10].

Characteristics of MSCs Relevant to Periodontal Regeneration

Multipotency: MSCs exhibit a remarkable property known as multipotency, which means they can differentiate into various cell types, making them exceptionally well-suited for regenerating periodontal tissues. In periodontal regeneration, this multipotency is crucial as it allows MSCs to give rise to cell types essential for restoring damaged structures within the periodontium [11]. MSCs can differentiate into osteoblasts, the cells responsible for bone formation, aiding in the regeneration of alveolar bone. Additionally, they can differentiate into chondrocytes, which play a role in cartilage formation, and adipocytes, which can contribute to fat tissue formation. This versatility is a crucial feature that positions MSCs as valuable tools in regenerating the diverse tissues of the periodontium [12].

Immunomodulatory effects: MSCs possess distinctive immunomodulatory properties that are of significant importance in the context of periodontal regeneration. The periodontal microenvironment is often characterized by chronic inflammation, driven by the host's immune response to microbial pathogens. MSCs can suppress this excessive inflammatory response, thus aiding in regulating the local immune environment. This anti-inflammatory effect is crucial for the success of regenerative therapies because persistent inflammation can hinder the healing and regeneration of periodontal tissues. MSCs can help temper the immune response and create a more favorable environment for tissue repair and regeneration [13].

Trophic and paracrine effects: MSCs secrete a diverse range of bioactive molecules, including growth factors and cytokines, which have trophic and paracrine effects on neighboring cells and tissues. These secreted factors play a pivotal role in promoting tissue repair and regeneration. Growth factors like vascular endothelial growth factor (VEGF), fibroblast growth factor (FGF), and transforming growth factor-beta (TGF-β) are involved in stimulating angiogenesis, which is essential for the formation of new blood vessels within regenerating tissues. Additionally, these factors can influence cell behavior, proliferation, and differentiation, contributing to the overall regenerative potential of MSCs. The trophic and paracrine effects of MSCs enhance the microenvironment for tissue repair, making them valuable agents in periodontal regeneration [14].

Low immunogenicity: MSCs have the advantage of being less likely to provoke an immune response when transplanted, making them suitable for allogeneic transplantation, where the stem cells are derived from a donor other than the recipient. The low immunogenicity of MSCs is attributed to their low expression of major histocompatibility complex (MHC) class I molecules and the absence of MHC class II molecules. This feature reduces the risk of graft rejection and enables the use of MSCs from healthy donors to treat individuals with periodontal issues. It provides versatility and flexibility in clinical applications and holds promise for allogeneic transplantation in regenerative periodontal therapies [15].

iPSCs and Their Potential Applications

Patient-specific therapy: iPSCs offer an exciting opportunity for personalized regenerative treatments in periodontal disease. iPSCs can be generated from a patient's somatic cells, such as skin or blood cells, through reprogramming. This reprogramming allows these iPSCs to exhibit pluripotency, similar to embryonic stem cells. As a result, iPSCs can be differentiated into cell types relevant to periodontal tissues, making it possible to develop patient-specific stem cell lines for regenerative therapies. This personalized approach can revolutionize periodontal treatments by tailoring the therapy to each patient's unique genetic background, potentially enhancing treatment effectiveness and outcomes [16].

Disease modeling: iPSCs provide a valuable platform for modeling periodontal diseases in vitro. By reprogramming somatic cells from patients with specific periodontal conditions, researchers can generate iPSCs that carry the genetic and cellular characteristics associated with those conditions. These iPSCs can be further differentiated into periodontal cell types, allowing researchers to study the disease at a cellular level. Disease modeling with iPSCs enables a better understanding of the underlying genetic and cellular mechanisms involved in periodontal diseases, facilitating the development of targeted therapies and potentially uncovering new treatment approaches [17].

Drug testing and screening: iPSCs open up possibilities for more effective drug development and testing in periodontal disease. By using patient-specific iPSCs, researchers can create disease models that closely mimic the individual patient's condition. This approach allows for the screening of potential drug candidates in a patient-specific context, providing a more accurate representation of how a drug may affect the patient's periodontal tissues. iPSC-based drug testing and screening can lead to the development of more effective therapeutic interventions personalized to the patient's needs and disease profile. This tailored approach can potentially optimize treatment outcomes while minimizing adverse effects, ultimately improving the management of periodontal diseases [18].

Mechanisms of action of stem cells in periodontal regeneration

Immunomodulatory Effects of Stem Cells in the Periodontal Microenvironment

Suppression of inflammation: MSCs possess a remarkable capability to suppress inflammation, making them valuable players in managing the chronic inflammatory environment associated with periodontal disease. Inflamed periodontal tissues often exhibit an overactive immune response to microbial pathogens, leading to tissue destruction. MSCs intervene by downregulating pro-inflammatory responses, dampening the release of inflammatory cytokines and chemokines, and modulating immune cells. This anti-inflammatory action is critical in mitigating chronic inflammation that contributes to the progression of periodontal disease. By reducing inflammation, MSCs create a more favorable microenvironment for tissue repair and regeneration, which is pivotal for successful regenerative therapies [19].

Regulatory T-cell (Tregs) induction: MSCs can promote the generation of Tregs, a subset of immune cells with a central role in maintaining immune homeostasis. Tregs are essential for preventing excessive immune responses that can lead to tissue damage and destruction. MSCs contribute to immune balance by inducing the generation and activation of Tregs within the periodontal microenvironment. Tregs function by suppressing the activity of pro-inflammatory immune cells, helping to prevent collateral damage to periodontal tissues. This regulatory function is instrumental in managing the immune response in the context of periodontal disease, preventing the unwarranted inflammation that can hinder the success of regenerative therapies [20].

Immune cell recruitment: MSCs serve a multifaceted role by not only suppressing inflammation but also by actively participating in the recruitment of immune cells to the site of injury within the periodontal tissues. This recruitment function aids tissue repair and wound healing by promoting a controlled and balanced immune response. MSCs can attract immune cells, such as macrophages and neutrophils, to the injured area. These immune cells play various roles in resolving inflammation and tissue repair. By orchestrating the controlled migration of immune cells, MSCs contribute to the overall regenerative potential of the periodontal microenvironment. This immune cell recruitment is crucial for creating a conducive environment for the repair and regeneration of periodontal tissues, which is central to the success of regenerative therapies [21].

**Figure 1:**
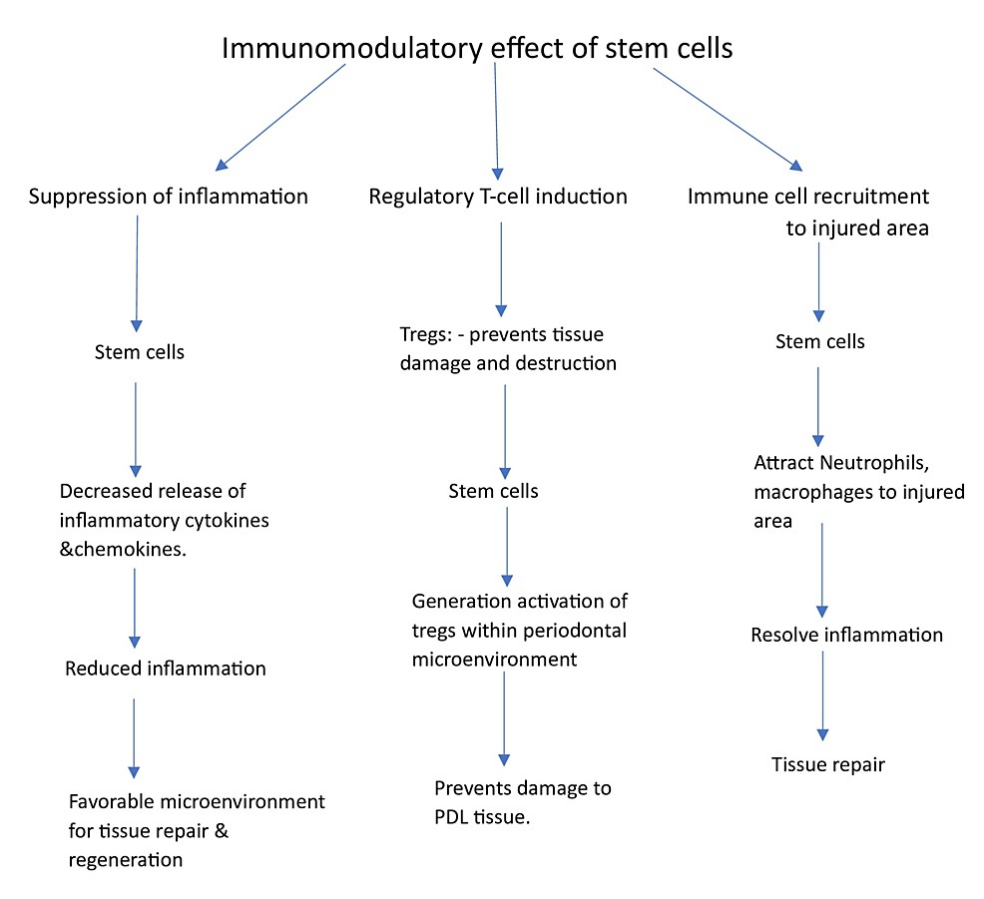
Mechanism of action of stem cells in periodontal regeneration Tregs: regulatory T-cells; PDL: periodontal ligament Image credit: Tanvi Bharuka

The mechanism of action of stem cells is summarized in Figure 1.

Differentiation Potential of Stem Cells Into Periodontal Tissues

Osteogenic differentiation: One of the critical mechanisms by which stem cells contribute to periodontal regeneration is through their ability to undergo osteogenic differentiation. Stem cells, particularly MSCs, can differentiate into osteoblasts, the specialized cells responsible for bone formation. This osteogenic potential is particularly vital for regenerating the alveolar bone, a critical component of the periodontium. The alveolar bone plays a pivotal role in supporting and anchoring teeth. Periodontal diseases often result in bone loss, which can lead to tooth mobility and loss. The differentiation of stem cells into osteoblasts facilitates the restoration of the alveolar bone, contributing to the overall success of periodontal regeneration [22].

PDL cell differentiation: Another essential aspect of stem cell-mediated periodontal regeneration is their potential to differentiate into PDL cells. The PDL is a specialized connective tissue that anchors teeth to the alveolar bone, providing stability and shock absorption during mastication. Stem cells, particularly those with multipotent properties, can differentiate into PDL cells. This differentiation process is crucial for restoring the PDL, ensuring that teeth are anchored and stable within the dental arch. By regenerating PDL cells, stem cell-based therapies aim to improve tooth stability and prevent further tooth mobility, a common consequence of periodontal disease [23].

Cementogenic differentiation: Some stem cell types possess the unique ability to differentiate into cementoblasts, specialized cells responsible for the formation of cementum. The cementum is a calcified tissue that covers the tooth's root surface and plays a role in tooth attachment to the PDL. Stem cell differentiation into cementoblasts is significant for periodontal regeneration, particularly in cases where the cementum has been damaged or lost due to disease. By regenerating cementum, stem cell-based therapies aim to enhance the integrity of the tooth-root interface, promoting proper tooth anchorage and periodontal health. This cementogenic differentiation capability is a critical component of comprehensive periodontal tissue restoration [24].

Paracrine Effects and Their Role in Tissue Regeneration

Growth factors and cytokines: Stem cells play a crucial role in promoting periodontal tissue regeneration by secretion of growth factors and cytokines. These bioactive molecules include VEGF, FGF, and TGF-β, among others. VEGF stimulates the formation of new blood vessels (angiogenesis), essential for delivering nutrients and oxygen to regenerating tissues. FGF promotes cell proliferation and tissue repair, while TGF-β involves various cellular processes, including tissue healing and immune regulation. By releasing these growth factors and cytokines, stem cells create a microenvironment conducive to tissue repair, angiogenesis, and overall regeneration within the periodontal region [25].

Extracellular vesicles (EVs): Stem cells release EVs, which are small membrane-bound structures containing microRNAs, proteins, and other bioactive molecules. These EVs play a pivotal role in cell-to-cell communication and can modulate the behavior of neighboring cells within the periodontal microenvironment. The microRNAs within EVs can influence gene expression in recipient cells, contributing to various cellular processes, including differentiation and proliferation. EVs from stem cells are actively involved in supporting tissue regeneration and maintaining a balanced immune response. Their role in intercellular communication helps orchestrate the regenerative processes, making them essential components of stem cell therapy in periodontal regeneration [26].

Tissue remodeling: The paracrine effects of stem cells extend to regulating extracellular matrix remodeling within the periodontal tissues. The extracellular matrix is the structural framework of tissues and plays a pivotal role in tissue architecture and functionality. Dysregulation of the extracellular matrix is common in periodontal diseases and can lead to tissue degradation. Stem cells, through their paracrine actions, help to maintain a balanced extracellular matrix by influencing the activity of cells involved in tissue remodeling. This regulation supports restoring tissue architecture, functionality, and homeostasis in the periodontal region, contributing to the overall success of regenerative therapies. By promoting tissue remodeling, stem cells facilitate the reconstruction of periodontal tissues, ultimately improving periodontal health and function [27].

Preclinical studies of stem cell therapy in periodontal regeneration

Animal Models Used in Preclinical Studies

Rodent models (e.g., rats and mice) are commonly utilized in preclinical studies of stem cell therapy for periodontal regeneration. Researchers can assess the safety, efficacy, and regenerative potential of stem cell-based interventions within a controlled environment. Additionally, rodent models are beneficial for investigating fundamental biological processes and mechanisms involved in periodontal regeneration. While they may not fully replicate the complexity of the human periodontal disease, they serve as critical initial platforms for assessing the feasibility of stem cell therapy [28].

Porcine models offer advantages in preclinical research due to their dental anatomy and physiology, which are more akin to those of humans. Pigs have a similar dentition, tooth-root structure, and periodontal architecture, making them valuable for studies that require a closer resemblance to human conditions. Porcine models provide insights into the clinical translation of stem cell therapy as they allow researchers to evaluate regenerative interventions in a setting that more closely mirrors the human periodontium. Their larger size and oral similarities offer a unique perspective on applying stem cell therapy in a more clinically relevant context, bridging the gap between rodent models and human trials [29].

Non-human primates, such as macaques, are used in some cases to study the effects of stem cell therapy in periodontal regeneration. These models offer distinct advantages due to their genetic and physiological similarities to humans. Non-human primates share significant genetic homology with humans, providing a relevant platform for assessing the safety and efficacy of stem cell therapies in a setting that closely resembles the human response. Their larger size and complex periodontal anatomy allow for a more comprehensive evaluation of stem cell-based interventions. While non-human primate models are typically reserved for advanced preclinical research, they can provide critical data on the potential clinical translation of stem cell therapy for periodontal disease in a way no other animal models can replicate [30].

Analysis Methodologies of Stem Cell Therapy in Periodontal Regeneration

Histological and radiographic analysis: To evaluate the effectiveness of stem cell therapy in periodontal regeneration, researchers employ histological and radiographic assessments. Tissue samples obtained from the treated areas, often through biopsy or extraction, are subjected to histological analysis. This involves microscopic examination of the samples to assess the regeneration of periodontal tissues, including alveolar bone, cementum, and PDL. In parallel, radiographic images, such as X-rays or cone-beam computed tomography (CBCT) scans, provide a non-invasive visualization of the treated area. Comparing these images with untreated controls allows researchers to quantify the extent of tissue regeneration and assess the overall success of stem cell therapy [31].

Functional assessments: A critical aspect of periodontal regeneration is restoring tooth stability and function. To assess this, researchers conduct functional tests, such as bite force analysis. These tests measure the strength of the bite and the ability of the treated teeth to withstand functional loads. Evaluating bite force provides insights into the practical implications of periodontal regeneration achieved through stem cell therapy. It helps determine whether the therapy not only restores the structure of periodontal tissues but also enhances the patient's ability to bite and chew effectively [32].

Immunohistochemical and molecular analyses: Researchers delve into the cellular and molecular mechanisms underlying stem cell therapy by performing immunohistochemical and molecular analyses. Immunohistochemistry involves staining tissue samples with specific antibodies to detect the presence and distribution of relevant markers and proteins associated with periodontal regeneration. Molecular analyses, such as gene expression studies, help identify changes in gene activity linked to regenerative processes. These analyses provide a deeper understanding of how stem cell therapy influences the biological aspects of periodontal regeneration and may reveal potential targets for therapeutic intervention [33].

Micro-computed tomography (micro-CT) imaging: Micro-CT imaging is a powerful tool for assessing changes in bone volume and architecture. It provides detailed three-dimensional images of the treated area, allowing for precise quantification of bone regeneration. Researchers can measure parameters such as bone volume, trabecular thickness, and bone density, offering a comprehensive view of the structural changes within the treated periodontal tissues. Micro-CT imaging is particularly valuable for assessing the efficacy of stem cell therapy in restoring bone architecture and volume [34].

Assessment of inflammation and immunomodulation: Stem cell therapy exerts immunomodulatory effects within the periodontal microenvironment. Researchers evaluate how much stem cell therapy influences inflammatory responses and the immune milieu in periodontal tissues. This assessment includes analyzing changes in the levels of specific cytokines, immune cell populations, and markers of inflammation. By understanding the impact of stem cell therapy on the local immune response, researchers can gain insights into the therapy's immunomodulatory effects, which are critical for managing chronic inflammation in periodontal disease and fostering tissue regeneration [35].

Challenges and Limitations Observed in Preclinical Studies

Heterogeneity of results: One of the primary challenges in interpreting preclinical studies of stem cell therapy in periodontal regeneration is the inherent heterogeneity in experimental conditions. Variations in factors such as the choice of animal models, the specific types of stem cells utilized, and the diverse disease models employed can result in inconsistent results. This heterogeneity can make it difficult to draw definitive conclusions and may require careful consideration when extrapolating findings to clinical applications. Establishing more standardized protocols and experimental conditions could address this issue and improve the reliability of preclinical research [36].

Limited clinical relevance: While preclinical studies provide valuable insights into the potential of stem cell therapy, the outcomes observed in animal models only sometimes directly translate to the clinical setting. Factors such as differences in anatomy, immune responses, and disease progression between animals and humans can limit the clinical relevance of preclinical findings. It is crucial to recognize that the success achieved in animal studies may only be partially replicable in human patients. To bridge this gap, thorough testing in clinical trials is essential to confirm the safety and efficacy of stem cell therapy in humans [37].

Long-term safety and efficacy: Preclinical studies often have relatively short follow-up periods, which may not capture the long-term safety and efficacy of stem cell therapy. While initial results may appear promising, potential adverse effects or the sustainability of regenerative outcomes over an extended period require further investigation. Long-term studies and continuous monitoring of patients who have undergone stem cell-based treatments are necessary to assess the durability and safety of these interventions [38].

Standardization of protocols: The need for standardized protocols for stem cell therapy in periodontal regeneration poses a significant challenge. Without consistent methodologies and treatment approaches, comparing results across different preclinical studies becomes problematic. Standardizing protocols for stem cell isolation, differentiation, administration, and monitoring would facilitate more robust and reliable research. These standardized procedures would enable researchers to better evaluate the safety and efficacy of stem cell therapy and facilitate the translation of research findings into clinical practice [36].

Regulatory and ethical considerations: Using stem cells, mainly derived from humans, in preclinical research raises regulatory and ethical concerns. Ensuring that stem cell research complies with existing regulations and ethical guidelines is paramount. The ethical use of human-derived stem cells and adherence to regulatory requirements, such as obtaining informed consent and rigorous oversight, are essential aspects of responsible stem cell research. Ethical and regulatory considerations are pivotal in advancing the field of stem cell therapy while maintaining the highest standards of research ethics and patient safety [39].

Clinical applications of stem cell therapy in periodontal regeneration

Clinical Trials and Case Studies Involving Stem Cell Therapy in Periodontal Regeneration

Overview of clinical trials: Clinical trials are a pivotal phase in the journey of stem cell therapy from preclinical research to real-world applications in periodontal regeneration. These trials involve human subjects and are designed to assess the safety and efficacy of stem cell-based interventions rigorously. This section provides a comprehensive overview of clinical trials investigating the use of stem cell therapy for periodontal regeneration. It outlines the key features of these trials, including the patient population, treatment protocols, and outcomes. Summarising the findings and experiences from these trials offers a critical perspective on the feasibility of stem cell therapy in a clinical context, shedding light on its potential benefits, limitations, and challenges [40].

Case studies: Case studies provide an in-depth look at the practical application of stem cell therapy in individual patients suffering from periodontal disease. Highlighting specific cases where stem cell therapy has been employed can offer valuable insights into its real-world impact on periodontal health. These case studies present detailed patient histories, treatment approaches, and the outcomes of stem cell therapy, illustrating the personalized nature of regenerative interventions. By showcasing the experiences and results of individual patients, this section serves to humanize the potential of stem cell therapy, demonstrating how it can significantly improve the quality of life for those affected by periodontal disease. Case studies offer a tangible and relatable perspective on the clinical utility of stem cell therapy in periodontal regeneration [41].

Assessment of Safety and Efficacy in Human Trials

Safety considerations: When evaluating the safety of stem cell therapy in the context of clinical trials for periodontal regeneration, it is essential to provide an in-depth analysis of the safety profile. This involves a detailed examination of any adverse events or side effects associated with the therapy. Adverse events can encompass a range of outcomes, from minor discomfort to more severe complications. It is crucial to not only report the occurrence of adverse events but also to assess their significance in the overall safety assessment. Factors such as the frequency, severity, and reversibility of adverse events play a role in determining the safety of the treatment. Additionally, the section should address the measures taken to monitor and mitigate potential risks, such as infection or allergic reactions, and how they contribute to ensuring the safety of stem cell therapy in the context of periodontal regeneration [41].

Efficacy outcomes: To assess the efficacy of stem cell therapy in clinical trials, it is imperative to analyze a range of outcomes comprehensively. This includes evaluating improvements in periodontal tissue regeneration, as measured through histological and radiographic assessments. Clinical parameters, such as changes in probing depth, attachment gain, and reductions in gingival inflammation, should also be examined. Additionally, patient-reported outcomes, such as improvements in quality of life and oral function, offer insights into the therapy's impact on the patient's well-being. The statistical significance of these results should be discussed, providing a quantitative assessment of the effectiveness of stem cell therapy. Comparing these outcomes with baseline measurements and control groups, if applicable, contributes to a comprehensive evaluation of the therapy's efficacy [40].

Long-term follow-up: The importance of long-term follow-up in assessing the durability of the therapeutic effects cannot be overstated. Stem cell therapy aims to provide lasting improvements in periodontal health and stability. Therefore, the section should address the need for extended monitoring of patients over an extended period to determine if the regenerative effects are maintained. This includes an evaluation of the longevity of improvements in clinical parameters, such as attachment gain and probing depth reduction. The section should discuss the implications of long-term follow-up in confirming the sustainability of periodontal regeneration achieved through stem cell therapy, emphasizing the potential for lasting benefits [40].

Comparison with standard treatments: Comparing the safety and efficacy of stem cell therapy with existing standard treatment modalities for periodontal disease is critical for understanding its potential advantages and limitations. This comparison should address how stem cell therapy measures against conventional approaches, such as scaling and root planing, guided tissue regeneration, or bone grafting. Highlighting the strengths and weaknesses of each treatment option in terms of safety and efficacy can guide clinicians and researchers in making informed decisions about the most suitable approaches for individual patients. Additionally, this section can offer insights into the potential of stem cell therapy to advance the field by providing novel benefits or improving upon existing standards of care [42].

Patient Selection Criteria and Treatment Protocols

Patient eligibility criteria: Factors considered when selecting patients may include disease severity, general health status, age, and other relevant parameters. For example, patients with moderate to severe periodontal disease or specific clinical indications may be eligible. General health assessments, such as the absence of contraindications to stem cell therapy, could also be part of the criteria. The section should provide a comprehensive overview of the patient selection process, highlighting the rationale for each criterion and its importance in ensuring the safety and efficacy of stem cell therapy [43].

Informed consent and ethical considerations: Ensuring ethical compliance and obtaining informed consent from patients are paramount when using stem cell therapy in clinical practice. This section should delve into the ethical considerations related to patient consent, emphasizing the need for patients to fully understand the nature of stem cell therapy, its potential risks and benefits, and the alternatives available. It should also discuss the responsibility of healthcare professionals in providing comprehensive information to patients to enable them to make informed decisions. Addressing ethical issues, such as the responsible use of human-derived stem cells and protecting patient rights, is essential. Compliance with ethical guidelines and regulations should be emphasized to maintain the highest ethical standards in clinical practice [44].

Treatment protocols: Detailing the protocols employed in clinical trials or clinical settings is crucial for understanding how stem cell therapy is applied. This should include information about the source of stem cells, such as whether they are derived from the patient's tissues or an allogeneic source. The section should also cover the specific methods and devices used for stem cell delivery, the timing of treatment (e.g., whether it is applied during surgical procedures), and any adjunctive therapies or medications used in combination with stem cell therapy. Providing a comprehensive overview of the treatment protocols allows readers to grasp the practical aspects of stem cell therapy implementation and highlights the methods used to optimize patient outcomes [45].

Challenges and ongoing research: Acknowledging the challenges and limitations encountered in clinical applications of stem cell therapy is essential. This section should discuss any hurdles in clinical practice, whether related to safety, efficacy, or other aspects of treatment. It should also address ongoing research efforts aimed at refining treatment protocols, enhancing patient outcomes, and addressing regulatory issues. This may include ongoing studies aimed at optimizing the selection of stem cell sources, improving delivery methods, or investigating the long-term safety and durability of therapeutic effects. Acknowledging these challenges and research endeavors demonstrates a commitment to continuous improvement and advancing stem cell therapy in periodontal regeneration [46].

Challenges and future directions

Regulatory Challenges and Ethical Considerations in Stem Cell Therapy

Regulatory framework: The section on the regulatory framework should provide an in-depth analysis of the existing regulations and guidelines governing the use of stem cell therapy in clinical practice. It should emphasize the critical importance of clear and comprehensive regulatory oversight to ensure patient safety and treatment efficacy. This involves discussing the role of regulatory bodies, such as the Food and Drug Administration (FDA) in the United States or the European Medicines Agency (EMA), in evaluating and approving stem cell therapies. The section should also highlight the necessity of obtaining the appropriate approvals and licenses for conducting clinical trials and applying stem cell-based treatments. Adhering to Good Manufacturing Practices (GMP) for stem cell production and processing should be emphasized. Addressing the regulatory framework underscores the commitment to maintaining the highest standards in stem cell therapy and ensuring that patients receive safe and effective treatments [47].

Ethical concerns: Ethical considerations related to the use of stem cells in clinical practice are of utmost importance. This section should delve into the ethical aspects of stem cell research and clinical applications, with a particular emphasis on the need for responsible research and patient consent. Discussing the ethical implications of using human-derived cells, protecting patient rights, and the responsible conduct of research is pivotal. It should also address ethical guidelines and principles, such as those outlined in the Declaration of Helsinki, that govern the ethical use of stem cells. Ensuring that patients are fully informed and provide informed consent for stem cell therapy is a fundamental ethical requirement. This section should underscore the ethical responsibility of healthcare professionals and researchers to uphold the highest ethical standards in stem cell therapy [48].

International collaboration: Recognizing the potential for international collaboration to establish harmonized standards and regulations for stem cell-based therapies is vital. This section should explore the benefits of cooperation among countries and regions to create a unified framework for the safe and ethical use of stem cell therapies. Discussing the role of international organizations, such as the World Health Organization (WHO) or the International Society for Stem Cell Research (ISSCR), in promoting international collaboration is essential. Emphasizing the need for sharing best practices, harmonizing regulations, and establishing common standards for stem cell therapy ensures that patients worldwide can access safe and effective treatments while maintaining ethical and regulatory compliance. International collaboration can accelerate the advancement of stem cell therapies and foster global innovation in the field [49].

Standardization of Protocols for Clinical Application

Protocol variability: Recognizing and addressing the variability in stem cell therapy protocols across different studies and clinical settings is critical. This section should underscore the challenges arising from the diversity in treatment approaches, including variations in stem cell sources, preparation methods, delivery techniques, and timing of treatment. The discussion should emphasize the potential consequences of this variability, such as difficulties in comparing results across studies and clinics and the potential impact on patient outcomes. Addressing the need for standardized treatment protocols to enhance consistency and comparability is pivotal. Highlighting the benefits of standardized protocols, such as improved treatment quality, reduced risk of adverse events, and more reliable research outcomes, underscores the importance of addressing protocol variability in stem cell therapy for periodontal regeneration [38].

Best practice guidelines: The section on best practice guidelines should stress the importance of developing standardized and evidence-based protocols that can serve as a reference for clinicians involved in administering stem cell therapy for periodontal regeneration. It should discuss the advantages of having clear guidelines, including improved treatment quality, enhanced patient safety, and increased confidence among healthcare professionals. The need for guidelines encompassing the selection of stem cell sources, treatment protocols, patient management, and follow-up care should be emphasized. Additionally, the role of professional organizations and international bodies in developing best practice guidelines can be discussed. These guidelines serve as a valuable resource for clinicians and researchers, guiding them in the responsible and effective use of stem cell therapy [50].

Collaboration with regulatory bodies: Addressing the collaboration between researchers, clinicians, and regulatory agencies is crucial for establishing standardized treatment protocols that adhere to safety and efficacy standards. This section should discuss how stakeholders can work together to align stem cell therapy protocols with regulatory requirements and expectations. Collaboration with regulatory bodies, such as the FDA in the United States or equivalent agencies in other regions, can help ensure that standardized protocols meet the necessary safety and efficacy criteria for approval and clinical practice. The section should also highlight the role of ongoing communication and cooperation with regulatory agencies to address evolving standards and regulations. Such collaboration contributes to establishing a robust and compliant framework for the responsible use of stem cell therapy in periodontal regeneration [51].

Prospects and Potential Advancements in the Field

Advanced cell therapies: The field of stem cell technology is rapidly advancing, with innovations such as iPSCs, gene-edited cells, and engineered cellular constructs holding great promise for periodontal regeneration. This section should delve into these cutting-edge developments, explaining how iPSCs, reprogrammed from adult cells to exhibit pluripotency, offer the potential for patient-specific regenerative treatments. Gene-edited cells, modified to express specific genes or functions, can play a role in enhancing regenerative capabilities. Engineered cellular constructs involving the manipulation of cell behavior and function have the potential to create specialized cell populations for improved periodontal tissue regeneration. Discussing the unique attributes and applications of these advanced cell therapies in the context of periodontal regeneration highlights their transformative potential in the field [52].

Personalized medicine: The concept of personalized regenerative medicine is reshaping how stem cell therapy is applied in periodontal regeneration. This section should explore the prospects of creating patient-specific stem cell lines tailored to individual needs and genetic backgrounds and discuss the methodologies for generating and utilizing patient-specific stem cells, highlighting the potential for customized treatments. The section should emphasize how personalized regenerative medicine can address individual variations in disease severity, tissue structure, and genetic factors, ultimately leading to more effective and patient-centered treatment approaches in periodontal regeneration [53].

Biomaterials and scaffolds: Biomaterials and scaffolds play an increasingly significant role in conjunction with stem cell therapy for periodontal regeneration. This section should highlight the integration of innovative biomaterials and scaffolds, which provide enhanced structural support and controlled release of growth factors to promote tissue regeneration. There is a need to discuss how these materials can mimic the natural microenvironment of periodontal tissues and support stem cell proliferation and differentiation. The potential for biomaterials and scaffolds to improve the efficiency and outcomes of stem cell therapy should be emphasized, as they enable a more controlled and precise approach to periodontal regeneration [54].

Immune modulation strategies: Ongoing research into modulating the immune response within the periodontal microenvironment holds great promise for optimizing tissue regeneration and minimizing inflammation. This section should examine the different explored approaches, such as immunomodulatory cytokines or Tregs induction, to fine-tune the immune response to better support the regenerative process. Additionally, a discussion is required on how these strategies aim to create a balanced and controlled immune environment that facilitates the success of stem cell-based therapies in periodontal regeneration. The section should emphasize their potential to address the chronic inflammation associated with periodontal disease and promote more effective tissue repair.

Translational research: Translational research is a pivotal bridge between preclinical studies and clinical practice. This section should explore the importance of translational research in facilitating the efficient integration of stem cell-based therapies into standard periodontal treatment protocols and discuss the steps in translating preclinical findings into clinical applications, highlighting the significance of rigorous testing, safety assessments, and regulatory compliance. It should also emphasize how translational research can expedite the clinical adoption of stem cell therapies, ultimately benefiting patients suffering from periodontal disease [55].

## Conclusions

In conclusion, the comprehensive review of stem cell therapy in periodontal regeneration has provided invaluable insights into a promising avenue for addressing the challenges of periodontal disease. Stem cell therapy, with its mechanisms of action involving immunomodulation, differentiation potential, and paracrine effects, offers a means to target the root causes of tissue destruction and chronic inflammation in the periodontium. Preclinical and clinical studies have demonstrated encouraging results, emphasizing the safety and efficacy of stem cell therapy while also underlining the need for standardized treatment protocols and rigorous regulatory and ethical considerations. The field is poised for advancements, such as personalized medicine and innovative biomaterials, offering tailored, more effective treatments for individuals suffering from periodontal disease. The potential impact on clinical practice is significant, promising more predictable and patient-specific regenerative outcomes while addressing the systemic implications of periodontal disease. Stem cell therapy represents a transformative approach to periodontal regeneration, offering new hope for improved patient well-being and an enhanced quality of life for individuals affected by this prevalent oral health concern. As research advances, maintaining a strong focus on regulatory compliance and ethical considerations is essential to ensure the responsible and evidence-based use of stem cell therapy in the dental profession.
